# Robust SiO_2_–Al_2_O_3_/Agarose Composite Aerogel Beads with Outstanding Thermal Insulation Based on Coal Gangue

**DOI:** 10.3390/gels8030165

**Published:** 2022-03-06

**Authors:** Jie Gu, Chao Ji, Rui Fu, Xin Yang, Zhichen Wan, Lishuo Wen, Qiqi Song, Yinghui Liu, Yaxiong Wang, Huazheng Sai

**Affiliations:** 1 School of Chemistry and Chemical Engineering, Inner Mongolia University of Science & Technology, Baotou 014010, China; gujie199504182021@163.com (J.G.); j1063708309@163.com (C.J.); yangxin975@163.com (X.Y.); wzc1866115326@163.com (Z.W.); a19819059052@163.com (L.W.); songqiqiaa@163.com (Q.S.); liuyinghui0419@163.com (Y.L.); wangyaxiong2021@126.com (Y.W.); 2 Inner Mongolia Engineering Research Center of Comprehensive Utilization of Bio-Coal Chemical Industry, Inner Mongolia University of Science & Technology, Baotou 014010, China; 3 Aerogel Functional Nanomaterials Laboratory, Inner Mongolia University of Science & Technology, Baotou 014010, China

**Keywords:** aerogel materials, composite materials, SiO_2_–Al_2_O_3_, coal gangue, mechanical properties, thermal properties

## Abstract

Advanced SiO_2_–Al_2_O_3_ aerogel materials have outstanding potential in the field of thermal insulation. Nevertheless, the creation of a mechanically robust and low-cost SiO_2_–Al_2_O_3_ aerogel material remains a considerable challenge. In this study, SiO_2_–Al_2_O_3_ aerogel based on coal gangue, which is a type of zero-cost inorganic waste, was constructed in porous agarose aerogel beads, followed by simple chemical vapor deposition of trimethylchlorosilane to fabricate SiO_2_–Al_2_O_3_/agarose composite aerogel beads (SCABs). The resulting SCABs exhibited a unique nanoscale interpenetrating network structure, which is lightweight and has high specific surface area (538.3 m^2^/g), hydrophobicity (approximately 128°), and excellent thermal stability and thermal insulation performance. Moreover, the compressive strength of the SCABs was dramatically increased by approximately a factor of ten compared to that of native SiO_2_–Al_2_O_3_ aerogel beads. The prepared SCABs not only pave the way for the design of a novel aerogel material for use in thermal insulation without requiring expensive raw materials, but also provide an effective way to comprehensively use coal gangue.

## 1. Introduction

Aerogels, miracle materials for the 21st century, are considered to be promising and useful because of their ultralow density (0.003–0.1 g/cm^3^), high porosity (80–99.8%), low thermal conductivity (0.013–0.14 W/m·K), high specific surface area (500–1200 m^2^/g), low dielectric constant (*k* = 1.0–2.0), and high acoustic impedance (~1.05 MRayl) [1,2,3]. Because of these excellent properties, aerogel materials have recently been the focus of considerable research and have been used in a wide range of applications such as thermal insulation [4,5,6], acoustic insulation [7], adsorption [8,9], flexible energy storage devices [10], and drug and catalyst carriers [11].

As a distinctive type of aerogel, SiO_2_–Al_2_O_3_ composite aerogel has better thermal stability than native SiO_2_ aerogels, which could make it suitable for use in aircraft [12,13]. Moreover, its unique three-dimensional network and nanoporous structure decreases heat conduction, so the thermal conductivity of the SiO_2_–Al_2_O_3_ aerogel is extremely low, allowing it to be used in insulation materials [14,15]. However, the high cost of the raw materials used to prepare this type of aerogel limits the extent to which its industrial production is feasible [15]. Preparation of the SiO_2_–Al_2_O_3_ composite aerogel with low-cost materials has attracted attention in recent years. Solid wastes such as fly ash, kaolin, and coal gangue have been considered to be raw material for preparing SiO_2_–Al_2_O_3_ composite aerogel due high silica and alumina contents. Shen et al. proposed synthesis of SiO_2_–Al_2_O_3_ composite aerogel from fly ash by alkali fusion-acid-leaching ambient-pressure drying process [14]. Moreover, Zhu et al. obtained the hydrophobic SiO_2_–Al_2_O_3_ composite aerogel from coal gangue [16]. Nevertheless, relevant reports still are rare. It is necessary to strengthen research in this area. As a hazardous solid waste, natural coal gangue produced during the excavation and washing processes of coal contains many useful minerals, such as superfine kaolin, aluminum oxide and silicon oxide [17]. In addition, scrapped coal gangue has caused significant environmental and economic problems in recent years. The current use of coal gangue mainly focuses on concrete, bricks, adsorbents, insulation panels and agricultural fertilizer [18]. Nevertheless, there is a lack of high-value applications for coal gangue. Hence, preparing SiO_2_–Al_2_O_3_ aerogel based on coal gangue is an environmentally friendly and cost-effective strategy.

In addition, the unique microstructure of inorganic oxide aerogels not only endows them with many excellent properties, but also inherently causes high brittleness, which greatly restricts their practical application [19,20,21]. The prepared aerogels also have the disadvantage of high brittleness in the report above. The fragility of aerogels can be traced to the necks of secondary particles, between which only a few chemical bonds exist [22,23,24]. To date, a variety of methods has been employed to improve the mechanical properties of inorganic oxide aerogels, including doping with nanofillers, employing a suitable precursor for cross-linking, and conformally coating the gel skeleton with a polymer [25,26,27]. All these methods improve the strength of the gel skeleton in the gelation process to mitigate the drawbacks of high brittleness of inorganic oxide aerogels. Nevertheless, this enhancement of mechanical properties of inorganic oxide aerogels using these methods comes at the cost of sacrificing other functionality, for example, by increasing thermal conductivity and density or decreasing porosity and specific surface area [28,29,30]. Worse still, in some cases, these modified hybrid inorganic oxide aerogels incorporated with polymers or nanofillers exhibit poor thermal stability because of the nature of organic or carbon-based fillers, making them unsuitable for thermal insulation [31]. Therefore, the development of a simple and convenient but feasible simple approach to enhance their mechanical properties is urgently needed.

**Figure 1:**
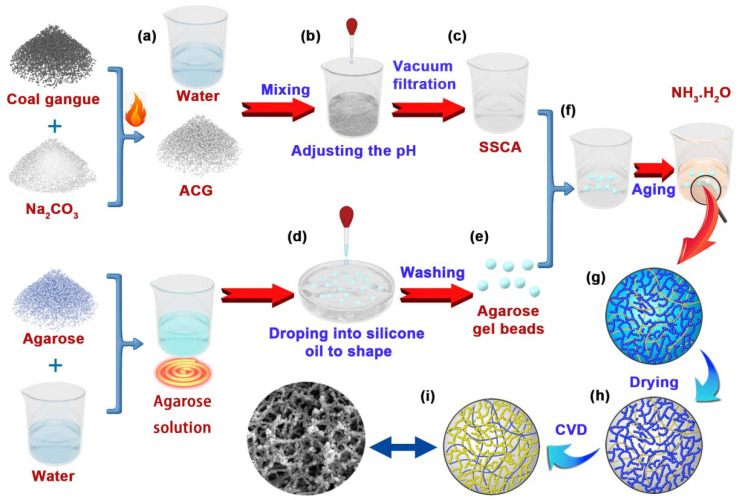
Schematic of the SCAB preparation process. (**a**) Activated coal gangue (ACG) is prepared by calcining a mixture of coal gangue and Na_2_CO_3_, then adding deionized water. (**b**) The pH of the suspension is adjusted. (**c**) SiO_2_ sol containing Al^3+^ (SSCA) is obtained by vacuum filtration. (**d**) Agarose wet gel beads formed via the agarose (a linear polymer with a long chain alternately linked to 1,3-linked β-D-galactose and 1,4-linked 3,6-endoether-L-galactose) solution are dropped into the silicone oil. (**e**) After washing, clean agarose wet gel beads are obtained. (**f**) The agarose wet gel beads are soaked in SSCA. (**g**) After aging and soaking in NH_3_·H_2_O solution, SiO_2_–Al_2_O_3_/agarose composite wet gel beads are obtained. (**h**) The wet gel beads are supercritically dried to obtain SCABs. (**i**) Hydrophobic SCABs are obtained via CVD process.

Herein, as shown in Figure 1, SCABs based on coal gangue of high source abundance were successfully synthesized by impregnating porous agarose aerogel beads with SiO_2_–Al_2_O_3_ aerogel. By adopting simple chemical vapor deposition (CVD) using trimethylchlorosilane (TMCS), the materials are given excellent hydrophobicity (approximately 128°). The unique nanoscale interpenetrating network structure endows the aerogel with excellent mechanical properties. Moreover, the unique structure preserves the high specific surface area (538.3 m^2^/g) of traditional aerogels, along with high porosity and remarkable thermal insulation properties. To the best of our knowledge, the aerogel beads prepared in this study are among the first to demonstrate effective performance in all these aspects without any major sacrifices. Therefore, these SCABs have both a low cost and outstanding mechanical properties, and are expected to be applied for use in thermal insulation.

## 2. Results and Discussion

### 2.1. Effect of Adding Water Volume to ACG on Gel Process

The main component of ACG is NaAlSiO_4_ (Figure S3) which can be decomposed in acid solution to obtain SSCA, but NaAlSiO_4_ requires sufficient time to be adequately decomposed. The SSCA was easily converted into gel before the ACG completely decomposed, because the direct addition of H_2_SO_4_ solution caused an exothermic reaction, increasing the temperature of the system. As a result, a large amount of residue remained in the reaction system. To reduce the generation of residue, adding a certain volume of deionized water to ACG and then dropping H_2_SO_4_ solution generated a concentration gradient in the solution, which prolonged the gelation time, effectively improving the extraction rate of silicon and aluminum elements.

**Figure 2:**
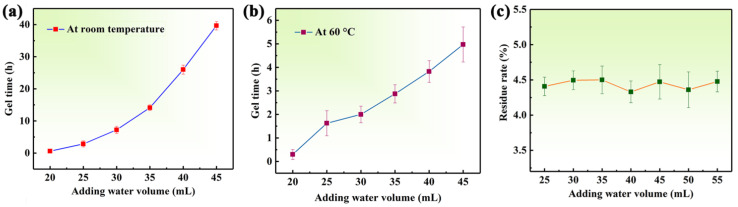
Effects of adding different volumes of water on (**a**) the gelation time at room temperature, (**b**) the gelation time at 60 °C, and (**c**) the residue rate.

The effects of adding amounts of water on the gelation time are shown in Table 1 and Figure 2a,b. It was found that the gelation time was shorter at 60 °C compared to room temperature when water was added the same volume. The reason for this could be understood to be that the silicate in SSCA was promoted faster, converting to SiO_2_ nanoparticles at high temperature. At the same time, the fast-moving SiO_2_ nanoparticles at high temperature easily collided with each other to form gel skeletons. Therefore, the gelation time was shorter at higher temperature. When 20 mL of deionized water was added to the ACG, the wet gel formed rapidly over a period of approximately 0.6 h at room temperature, which resulted in a large amount of residual ACG substance that was not decomposed embedded in the translucent gel as shown in Figure S4a. This was attributed that the water volume was too small to effectively alleviate rapid temperature rise resulting from releasing heat of the H_2_SO_4_ solution in contact with the ACG. When the added water volume reached 25–45 mL, the gelation time of samples all exceeded 1.0 h at room temperature, which enabled the ACG to be adequately decomposed in the acid solution as shown in Figure S4b. Meanwhile, increasing the volume of deionized water resulted in an increase in the gelation time (see Figure 2a,b). This could show that adding more water reduced the concentration of silica precursor (i.e., silicate in SSCA) and efficiently limited the obvious increase in temperature in the system resulting from the neutralization reaction of H_2_SO_4_ solution. If the added water volume were greater than 50 mL, the SSCA would also not gel, because the concentration of the silica precursor was relatively low and could not form a three-dimensional network gel skeleton, either at room temperature or 60 °C. The residue rate changed only minimally with increasing water volume, as shown in Table 1 and Figure 2c. In this situation, it can be speculated that the ACG decomposition is primarily related to the concentration of H^+^; all samples would eventually to be adjusted pH = 2 (25–50 mL), which ensured sufficient amount of acid to decomposed ACG.

### 2.2. Structural Determination of SCABs

**Figure 3:**
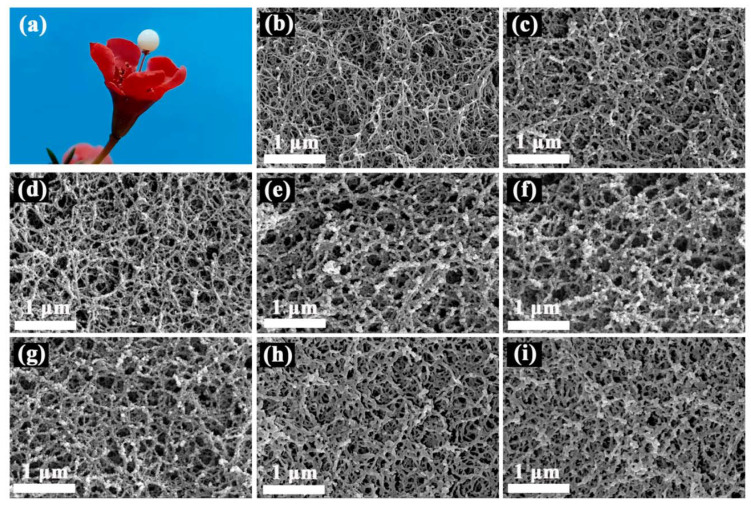
(**a**) Camera photo of the SCAB on flowers. SEM images of (**b**) AABs, (**c**) SCAB-1, (**d**) SCAB-2, (**e**) SCAB-3, (**f**) SCAB-4, (**g**) SCAB-5, (**h**) SCAB-6, and (**i**) SCAB-7.

The obtained SCABs presented uniform white sphericity with a diameter of 3 mm when placed in a culture dish as shown in Figure S5. The hydrophobic SCAB could remain on the slender pistil of the wintersweet flower, indicating promising lightweight properties due to its unique microstructure (Figure 3a). SEM images of the samples fabricated by adding different volumes of water to ACG were obtained to confirm how the concentration of SSCA influenced the morphology of the nanoscale interpenetrating network structure, which was formed by the interpenetration of a rigid SiO_2_–Al_2_O_3_ gel skeleton and a flexible agarose network gel skeleton. Native agarose aerogel beads (AABs) prepared from a 2 wt.% of agarose solution exhibited loose threadlike structures (Figure 3b). When a relatively high concentration (adding 25 or 30 mL of water to ACG) of SSCA was used to soak agarose wet gel beads to obtain samples, the threadlike structures of agarose and lesser three-dimensional network structure of SiO_2_–Al_2_O_3_ aerogel can be clearly observed in these SCABs (SCAB-1 and SCAB-2 in Figure 3c,d; please refer to the experimental section for naming). This could be understood as SiO_2_–Al_2_O_3_ composite nanoparticles being prone to agglomeration at high SSCA concentrations, resulting in relatively large nanoparticles, which have difficultly diffusing into the agarose network. When the concentration of SSCA was further decreased (adding 35 to 45 mL of water to ACG), a denser three-dimensional framework structure consisting of necklace-like connections of SiO_2_–Al_2_O_3_ composite nanoparticles were formed (Figure 3e–g), which corresponded to more SSCA entering the wet gel beads. Furthermore, the interpenetrating network structure composed of the gel skeleton formed by the SiO_2_–Al_2_O_3_ aerogel and the agarose aerogel can also be more clearly observed. A further decrease in the concentration of SSCA (adding 50 to 55 mL of water to ACG) showed a filamentous structure of SCAB 6-7 that was thicker than that of the AABs, whereas a SiO_2_–Al_2_O_3_ three-dimensional network structure was not observed (Figure 3b,h,i). Two possible explanations could account for this phenomenon. Above all, as the concentration of the SSCA gradually decreased, and SCAB-6 and SCAB-7 had the lower precursor concentration of SiO_2_–Al_2_O_3_ gel skeleton. Hence, most of the nanoparticles simply adhered to the matrix in the role of hydrogen bonds without forming an effective three-dimensional network structure. In addition, partial Al^3+^ partially entered the agarose wet gel and was soaked in NH_3_.H_2_O to form an Al(OH)_3_ wet gel that covered the surface of the agarose fibrous gel skeleton. Therefore, the interiors of SCAB-6 and SCAB-7 formed thicker filamentous microstructures.

**Figure 4:**
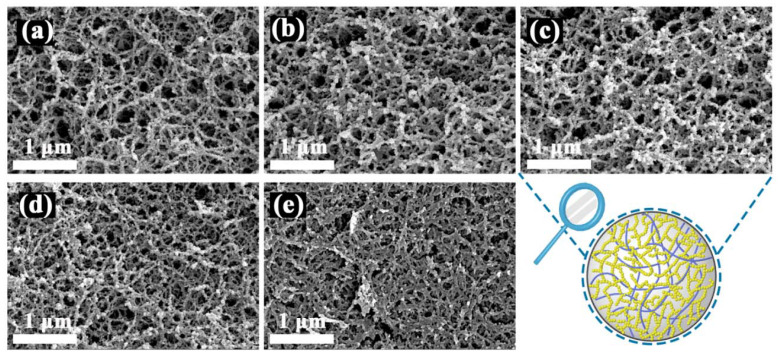
SEM images of (**a**) SCAB-8, (**b**) SCAB-9, (**c**) SCAB-10, (**d**) SCAB-11, (**e**) SCAB-12.

The morphology of SCABs showed different structures as the content of agarose changed (Figure 4). The agarose concentration of the SCABs affected the amount of SiO_2_–Al_2_O_3_ aerogel formed. When a relatively low concentration (1 wt.%) of agarose solution was used to construct SCAB-8 (Figure 4a), loose filamentous agarose network structures with large pore sizes were observed. Furthermore, sparse and thin SiO_2_–Al_2_O_3_ gel skeleton structures were also observed. As the agarose concentration increased (i.e., 1.5 and 2 wt.%), the agarose and SiO_2_–Al_2_O_3_ gel skeletons became denser (Figure 4b,c). This can be attributed to the fact that the partial SiO_2_–Al_2_O_3_ gel skeleton needs to attach to the agarose gel skeleton with a threadlike structure. However, when the concentration of the agarose solution increased to 2.5 and 3 wt.%, it was difficult to observe the SiO_2_–Al_2_O_3_ gel skeleton (Figure 4d,e). These phenomena can be ascribed to the fact that the agarose gel skeleton structures are denser, which limited SiO_2_–Al_2_O_3_ composite nanoparticles possessing a contain size due to the smaller pore of agarose gel surface entering the agarose beads inside.

**Figure 5:**
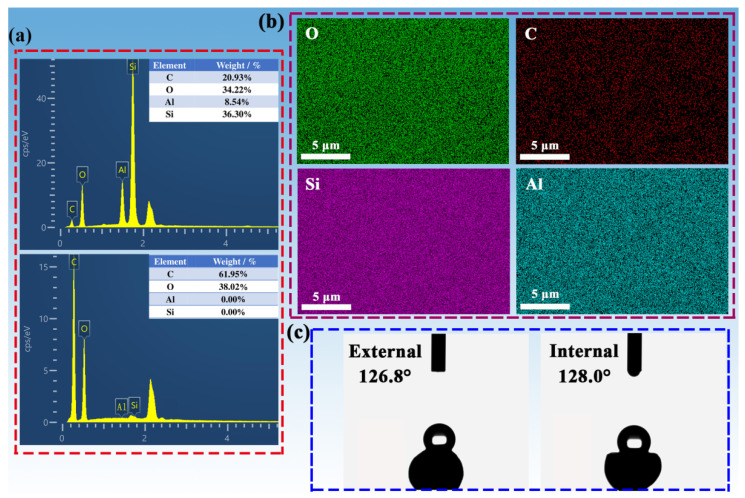
(**a**) EDS spectra of SCAB-4 and AAB with weight concentration for C, O, Si and Al. (**b**) EDS elemental (C, O, Si and Al) mapping images of SCAB-4. (**c**) External and internal water contact angles of SCAB-4.

The weight concentration of C, O, Si, and Al atoms were clearly observed in the EDS spectrum of SCAB-4, and the combined weight concentration of Si and Al was determined to be 44.84% (Figure 5a). However, the AAB contained no Si and Al elements. These phenomena indicate that the silica and alumina elements were successfully composited with agarose. From the element-mapping distribution (Figure 5b), it can be seen that the distribution of Si and Al in SCABs was relatively uniform. Hence, these phenomena indicated that the SiO_2_–Al_2_O_3_ aerogel was evenly composited in the SCABs in this synthesis method. Notably, the gel skeleton of the hydrophobic aerogel material would not be as easily destroyed by water vapor in the air, expanding its application [32]. As shown in Figure 5c the successful silane modification in this work could be confirmed from the contact angle test results, which showed internal and external contact angles above 125°. Thus, the SCABs possess wonderful hydrophobicity.

### 2.3. Nitrogen Adsorption–Desorption Test of SCABs

**Figure 6:**
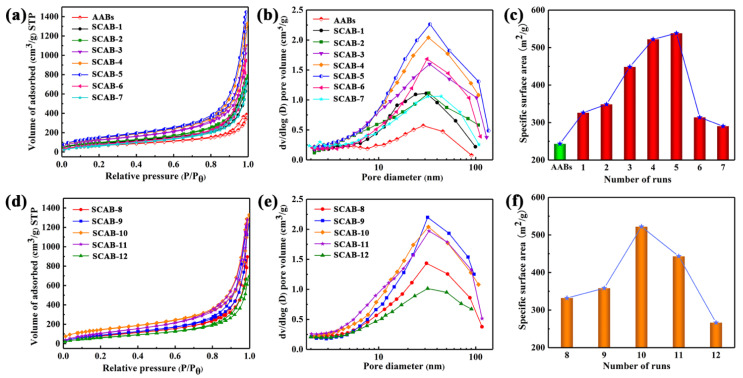
Nitrogen adsorption–desorption isotherms of AABs and SCAB 1–7 (**a**) and SCAB 8–12 (**b**). Pore-size distribution curves of AABs and SCAB 1–7 (**c**) and SCAB 9–12 (**d**). Rules of specific surface area change in AABs and SCAB 1–7 (**e**) and SCAB 9–12 (**f**).

The nitrogen adsorption–desorption curves and pore-size distribution curves, as well as the specific surface area of SCABs with different adding water volumes added to ACG and agarose solution concentrations, are shown in Figure 6. The hysteresis loops of SCABs, which existed in the nitrogen adsorption–desorption isotherm, were type-IV isotherms, representing mesoporosity [33]. Compared with AABs, the nitrogen adsorption and the relative Brunauer–Emmett–Teller (BET) surface area (Figure 6a,c) of the SCABs were higher. This phenomenon indicates that the SiO_2_–Al_2_O_3_ gel skeleton can offer a more mesoporous structure for the composite materials. With a decrease in the concentration of SSCA, the nitrogen adsorption amount and hysteresis loop present a trend that first increases and then decreases. In addition, the pore-size distribution studies showed that SCAB-4 and SCAB-5 had more obvious characteristics of mesoporous structures compared to other samples, which were prepared using the same concentration of agarose solution for SCAB-1 through SCAB-7. This was attributed to the greater formation of SiO_2_–Al_2_O_3_ gel skeletons because more SSCA entered the wet agarose gel beads when SSCA was at the proper concentration. Remarkably, a BET test showed that the samples had an extensive range of specific surface areas, from 290.3 m^2^/g to 552.1 m^2^/g (Figure 6c), with different SSCA concentrations. This is also because the concentration of SSCA has an important impact on the SiO_2_–Al_2_O_3_ gel skeleton.

With the increase in the concentration of agarose solution (1–2 wt.%) for preparing SCABs, the nitrogen adsorption capacity and specific surface area showed an increasing trend, and the mesoporous structure of SCAB 8–10 gradually became apparent (Figure 6d,e). This was attributed to the fact that agarose wet gel prepared from a solution with a higher concentration possessing a more fibrous gel skeleton enabled a certain number of SiO_2_–Al_2_O_3_ nanoparticles to be tightly deposited onto these gel skeletons. Nevertheless, when the agarose concentration was relatively high (2.5 and 3 wt.%), the nitrogen adsorption capacity and specific surface area decreased because the agarose wet gel formed a denser fibrous gel skeleton, making it difficult for SSCA to enter the agarose wet beads. The above results agree well with the characteristics of the SEM images described above (Figure 4).

The average desorption pore size (13.4 to 18.0 nm) of the samples is presented in Table 2. The BET surface area and average pore size showed the same tendency (Figure 6c,f). This phenomenon could be explained by the fact that when the content of SiO_2_–Al_2_O_3_ aerogel was relatively low, there were not enough SiO_2_–Al_2_O_3_ gel skeletons inside the agarose aerogels or the gel skeletons were not robust enough to resist the shrinkage of varying degrees of the samples during drying. Therefore, samples with low SiO_2_–Al_2_O_3_ aerogel content have smaller specific surface area and smaller average pore size. On the contrary, the samples exhibited a more obvious mesoporous structure (e.g., SCAB 4–5 and SCAB-11) and have a high specific surface area when the content of SiO_2_–Al_2_O_3_ aerogel in samples was relatively high.

### 2.4. Compression Test

**Figure 7:**
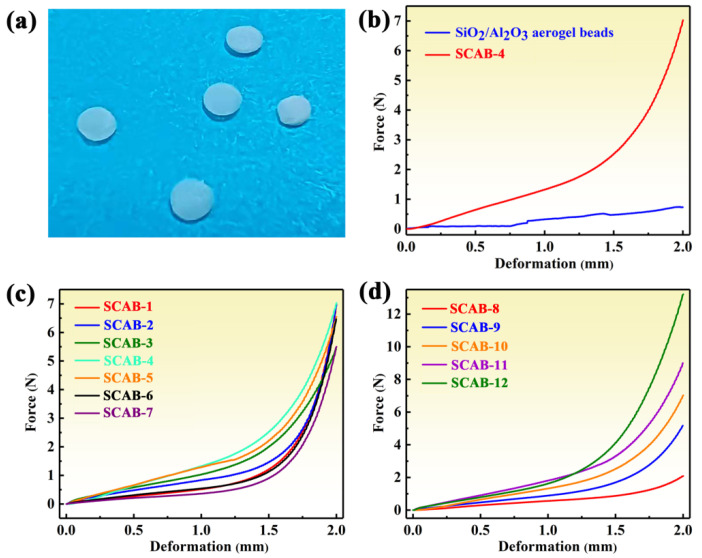
(**a**) Photos of SCABs after compression test. Compressive force–deformation curves of (**b**) native SiO_2_–Al_2_O_3_ aerogel beads and SCAB-4, (**c**) SCAB 1–7, (**d**) SCAB 8–12. The shape of the samples is spherical, which causes the cross-sectional areas to change continuously during the compression process, so the x- and y-axes in (**b**–**d**) are respectively represented by deformation and force.

Outstanding mechanical properties are essential for the transportation, installation, and use of thermal insulation materials [34]. Interestingly, fragile SiO_2_–Al_2_O_3_ aerogels combined with very excessively soft agarose aerogels could produce SCABs with an excellent flexibility and resistance to compression. Compression testing was performed to explore the mechanical properties of the SCABs. The force–deformation curve of native SiO_2_–Al_2_O_3_ aerogel beads presented an obvious zigzag shape, while SCAB-4 not only showed a smoother curve but also an increased compressive force (Figure 7b). This could be because the native SiO_2_–Al_2_O_3_ aerogel beads were easily broken into fragments when external pressure was applied, while SCAB-4 could maintain its integrity under pressure as shown in Figure 7a. Therefore, the native SiO_2_–Al_2_O_3_ aerogel is extremely fragile and cannot be used directly as a thermal insulation material. As shown in Figure 7c, the compressive force of SCABs first increased and then decreased before the deformation of the sample reached 1.3 mm. Nevertheless, the difference in the force required to compress the SCABs became small at a deformation of 2 mm. This was related to the formation of the rigid SiO_2_–Al_2_O_3_ gel skeleton within the space of the agarose network gel skeleton due to the ability of the three-dimensional skeleton network to resist compressive force. Therefore, SCAB 3–5, which contained more gel skeletons, was compressed, requiring a higher force than the other samples. This further proved the effective formation of a nano-interpenetrating network. However, when the compressive force reached a certain level, the three-dimensional framework of SiO_2_–Al_2_O_3_ aerogel collapsed. Introducing SiO_2_–Al_2_O_3_ aerogel insignificantly increased the solids content of SCABs due to SiO_2_–Al_2_O_3_ aerogel ultra-porous structure. Therefore, the difference in the compressive force became insignificant and reached 2 mm deformation.

The effect of agarose concentration on the compressive strength of SCABs was further studied. With the increase of agarose concentration, the difference of compressive force of the SCABs was relatively small before the deformation reached 1.3 mm, whereas the difference of compressive force of the samples significantly increased when the deformation reached 2.0 mm. This indicates that a large amount of fibrous agarose gel skeleton was formed with the increase of agarose solution, which contributed to effectively increasing the solid content of the SCABs (Figure 4) resulting in excellent compressive strength of the aerogels.

### 2.5. Thermal Stability

**Figure 8:**
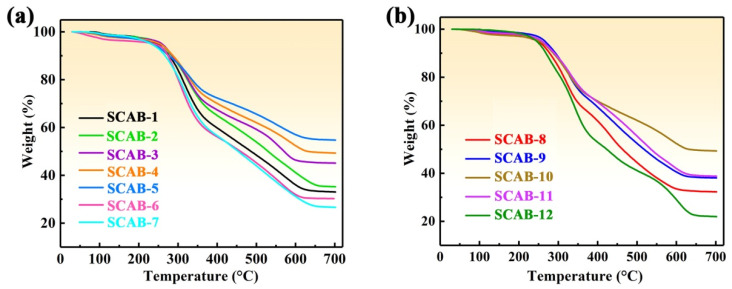
(**a**) Thermogravimetric analysis (TGA) curve of SCAB 1–7. (**b**) TGA curve of SCAB 8–12.

Because excellent thermal stability plays a key role in applications of composite materials [35], the thermal stability of samples was determined by TGA analysis in air, as shown in Figure 8. The thermal degradation of SCAB 1–12 took place in three steps similar weight-loss steps: 30–250 °C, 250–620 °C, and 620–700 °C (Figure 8). In the first stage (30–250 °C), degradation resulted from the volatilization loss of physically weak materials on the surface and evaporation-based loss of water vapor from the pores. The main degradation of SCABs occurred in the second stage from 250 to 620 °C, originating from the combustion of the agarose skeleton in the SCABs [36,37]. Moreover, the SCABs underwent hydrophobic treatment. Hence, their surfaces had significant amounts of –Si–O–CH_3_, which was degraded when the temperature exceeded 400 °C [38]. In the third stage (620–700 °C), there was no significant weight loss, indicating that the agarose and methoxy groups were exhausted. As shown in Figure 8a, the residue at 700 °C with decreasing SSCA concentration was observed to first increase (SCAB 1–5) then decrease (SCAB 5–7). In addition, the residue of SCABs at 700 °C significantly first increased (SCAB 8–10) and then decreased (SCAB 10–12) with increasing concentration of aerogel solution (Figure 8b). Given that agarose is a biopolymer that is degraded at high temperature while SiO_2_–Al_2_O_3_ aerogel is retained, the proportion of SiO_2_–Al_2_O_3_ aerogel in SCAB first increased (SCAB 1–5 and SCAB 8–10) then decreased (SCAB 5–7 and SCAB 10–12). This conclusion is consistent with the abovementioned conclusions of SEM and BET analysis of the SCABs (Figure 3, Figure 4 and Figure 6). In conclusion, the composite materials are suitable for use below 250 °C.

### 2.6. Thermal Transport Properties of SCABs

**Figure 9:**
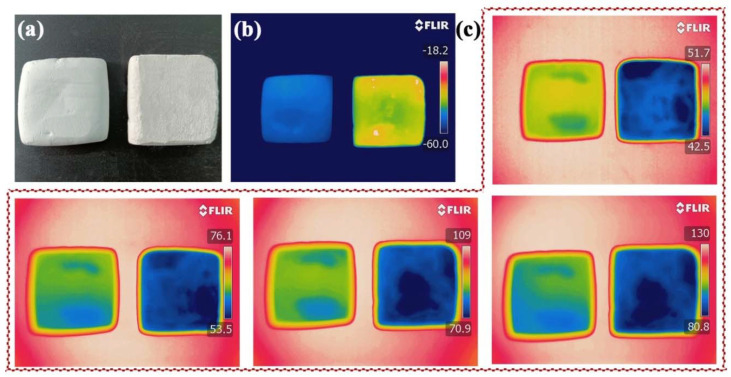
(**a**) Optical photo NPB and PBCS. FLIR images of the NPB and PBCS (**b**) on carbon ice base plate, and (**c**) heating base plates at different temperatures. (The unit of the temperature scale bar on the right of (**b**,**c**) is °C, and the FLIR images were automatically generated by the FLIR).

As aerogels are generally used for thermal insulation because of their light weight and highly porous internal structures [39], the thermal insulating performance of the aerogel was further evaluated. The influence of SCAB-4 in inhibiting heat transfer was demonstrated by placing a pure putty block (PPB) and putty block containing SCABs (PBCS) with similar thicknesses (7 mm) on either a cold (carbon dioxide ice cube at −60 °C as shown in Figure 9b) or hot plate with different temperatures, as shown in Figure 9c. The heat transfer through the putty blocks was visualized using an infrared camera (FLIR) on top of a hot surface when the temperatures of the samples remained constant for approximately 15 min. As shown in Figure 9b,c, the PBCS effectively blocked heat/cold transfer. At the same time, the difference in temperature on the hot and cold surfaces became more obvious as the base plate was kept away from room temperature. This indicates that heat was effectively blocked by the SCABs contained in the putty. Thus, it was shown that SCABs have excellent thermal insulation properties.

## 3. Conclusions

In conclusion, SCABs with unique nanoscale interpenetrating network structures based on coal gangue were easily synthesized by directly diffusing SSCA into a loose agarose gel skeleton. The properties of the obtained SCABs could be adjusted by changing the volume of water added to the ACG and the concentration of the agarose solution. Based on the above analysis of the microstructure and EDS of SCABs, it could be proved that the obtained samples exhibited a unique nanoscale interpenetrating network structure which was formed by the construction of rigid SiO_2_–Al_2_O_3_ gel skeleton in the void space of the flexible agarose gel skeleton network. Moreover, the SCABs exhibited high specific surface area (538.3 m^2^/g) and hydrophobicity (approximately 128°), as well as excellent thermal stability, and mechanical and thermal insulation properties. These characteristics mean that the SACBs are wonderful candidates for the applications of thermal insulation, such as coatings for exterior wall materials in modern buildings and pipelines for transporting liquid or gas. In addition, these SCABs pave the way for the design of novel aerogel materials in the field of thermal insulation without requiring expensive raw materials, but also provide a new direction for the comprehensive use of coal gangue.

## 4. Materials and Methods

### 4.1. Materials

Raw coal gangue from Tumd Right Banner in Baotou (Inner Mongolia, China) was crushed and sieved to 100 mesh size (150 μm). Agarose (≥97.0%) was purchased from Gene Technology Co., Ltd. (Hong Kong, China). Ethanol (≥99.7%) and commercial sodium carbonate (Na_2_CO_3_) were purchased from Sigma–Aldrich Chemical Ltd. (Darmstadt, Germany). Hydroxy silicone oil (100.0 mm^2^/s, viscosity) was supplied by Guanhua Trade Ltd. (Nanjing, China). Trimethylchlorosilane (TMCS, ≥98%), concentrated sulfuric acid (H_2_SO_4_) and ammonia (NH_3_·H_2_O) were purchased from Demont Chemical Ltd. (Nanjing, China).

### 4.2. Activation of the Coal Gangue

The coal gangue used in this experiment is mainly composed of SiO_2_ and Al_2_O_3_, which exist as kaolinite (Figure S1 and Table S1). These compounds are chemically stable and need to be activated for further use. The ACG was performed by following a previously reported similar method [14,17,40]. Please refer to the Supporting Information for further details.

### 4.3. Preparation of the SSCA

The ACG (5 g) was ground into a fine powder. Then, the powder was mixed evenly with different volumes of deionized water (see Table 3, Figure 1a), and the pH was adjusted to 2 using a 6 mol/L H_2_SO_4_ solution (Figure 1b). Subsequently, the mixture was stirred using a magnetic stirrer for 1 h to ensure that the ACG was completely dissolved in the acid solution. Finally, SSCA was obtained via a vacuum filtration process (Figure 1c) and the filtered residue was dried to calculate the residue rate (residue rate = drying residue mass/ACG mass × 100%). The SSCA contained in some impurities, such as Na^+^, SO_4_^2−^, and excess H^+^. Therefore, these impurities will be removed by an ethanol solution replacement process afterwards.

### 4.4. Preparation of the Agarose Gel Beads

Agarose solutions with different concentrations (see Table 3) were added to the injection pump (ISPLab01, Shenzhen, China), and then dropped into the hydroxy silicone oil at a rate of 3 mL/min (Figure 1d). After gelation of the agarose, the wet gel beads with a diameter of approximately 3.0 mm were obtained. Subsequently, the silicone oil on the surface of the gel beads was washed off with deionized water (Figure 1e).

### 4.5. Preparation of the SCABs

Agarose gel beads were completely impregnated in SSCA for 2 h at room temperature (wet gel bead mass remained unchanged), after which the gel beads were placed in the silicone oil at 60 °C for 6 h until the silicate in the agarose matrix was transformed into a silica gel skeleton (Figure 1f). Subsequently, the gel beads were transferred to the ammonia (1 mol/L) for 2 h to enable adequately form the Al(OH)_3_ gel at room temperature (Figure 1g). By employing the ethanol solution replacement method and supercritical drying process, SCABs were obtained (Figure 1h). Finally, the hydrophobic SCABs were produced by the CVD of TMCS (Figure 1i). Beakers with TMCS and SCABs were placed in an airtight mold at room temperature for 8 h. Then, SCABs modified with TMCS were placed in an oven at 50°C for 1.5 h to remove excess TMCS.

### 4.6. Preparation of the PBCS

Putty powder (10.0 g) and water (4 mL) were mixed evenly to obtain a putty paste. Next, SCABs (0.5 g) were added to the putty paste, stirred uniformly, and shaped into blocks of 7 mm thickness (the length and width were 30 mm and 25 mm, respectively). Finally, the wet putty blocks were dried in oven at 50 °C for 2 h to obtain the PBCSs. (The preparation of pure putty blocks (PPBs) is described in the Supporting Information.)

### 4.7. Characterization

The X-ray fluorescence (XRF) and X-ray diffraction (XRD) analysis were performed to characterize the major elements and phase of the coal gangue. Morphology and nanostructure of the related samples were analyzed. The energy dispersive spectrum (EDS), contact angles, specific surface area, pore-size distribution and the mechanical properties and chemical construction were determined. Finally, the thermos-gravimetry analysis (TGA) and thermal insulation performance of the prepared materials were evaluated. Detailed characterization methods are provided in the Supplementary Materials.

## Figures and Tables

**Table 1:** Effect of adding different volumes of water to ACG on gel process.

**Added Water Volume (mL)**	20	25	30	35	40	45	50	55
**Gelation Time (h) at Room Temperature**	0.6	2.8	7.2	14.1	26.0	39.7	—	—
**Gelation Time (h) at 60 °C**	0.30	1.6	2.0	2.9	3.8	5.0	—	—
**Residue Rate (%)**	—	4.41	4.49	4.50	4.33	4.47	4.36	4.48

**Table 2:** The average desorption pore size of the samples.

Sample (SCAB-)	1	2	3	4	5	6	7	8	9	10	11	12
Average pore size (nm)	14.6	15.4	16.2	17.6	18.0	14.3	13.4	15.6	16.5	16.8	15.5	13.5

**Table 3:** Volumes of deionized water added to prepare ACG and concentrations of agarose solutions used in each sample are shown in Table 3.

Sample	SCAB-1	SCAB-2	SCAB-3	SCAB-4	SCAB-5	SCAB-6	SCAB-7	SCAB-8	SCAB-9	SCAB-10	SCAB-11	SCAB-12
Added water volume (mL)	25	30	35	40	45	50	55	40	40	40	40	40
Agarose (wt. %)	2	2	2	2	2	2	2	1	1.5	2	2.5	3

## Data Availability

The data presented in this study are available on request from the corresponding author.

## Supplementary Materials

The following are available online at https://www.mdpi.com/article/10.3390/gels8030165/s1, Table S1: Elemental analysis of raw coal gangue by XRF; Figure S1: the major elements and phase of the raw coal gangue; Figure S2: The reaction rate of coal gangue under different parameters; Figure S3: XRD patterns of the ACG; Figure S4: The effect of adding water volume on gel. (a) Wet gel formed after adding 20 mL water into ACG. (b) The suspension was formed after adding 40 mL water into ACG. (c) SSCA was obtained by filtering the suspension. (d) A little residue on filter paper. (e) The gel formed from SSCA; Figure S5: Morphology photos of SCABs; Figure S6: FTIR spectra of samples; Figure S7: XRD patterns of the SCAB-4 [41]; Activation procedure of the coal gangue; The preparation of pure putty blocks (PPBs); Characterization and Reference.
