# Supplementary material for: Robust SiO2–Al2O3/Agarose Composite Aerogel Beads with Outstanding Thermal Insulation Based on Coal Gangue
Source: Gels. 2022 Mar 6;8(3):165. doi: 10.3390/gels8030165 (PMC8952686; doi:10.3390/gels8030165)
Supplement: Supplementary file 1 [file gels-08-00165-s001.zip › gels-1599545-supplementary.pdf]

gels

Supplementary

Robust SiO_2-Al_2O_3/agarose composite aerogel beads with outstanding thermal insulation based on coal gangue

Jie Gu [1,2,3,†], Chao Ji [1,2,†], Rui Fu [1,2,3,*], Xin Yang [1,2,3], Zhichen Wan [1,2], Lishuo Wen [1,2], Qiqi Song [1,2,3], Yinghui Liu [1,2], Yaxiong Wang [1,2,3] and Huazheng Sai [1,2,3,*]

1 School of Chemistry and Chemical Engineering, Inner Mongolia University of Science & Technology, Baotou 014010, China; gujie199504182021@163.com (J.G.); j1063708309@163.com (C.J.); yangxin975@163.com (X.Y.); wzc1866115326@163.com (Z.W.); a19819059052@163.com (L.W.); songqiqiaa@163.com (Q.S.); liuyinghui0419@163.com (Y.L.); wangyaxiong2021@126.com (Yaxiong Wang)
2 Inner Mongolia Engineering Research Center of Comprehensive Utilization of Bio-Coal Chemical Industry, Inner Mongolia University of Science & Technology, Baotou 014010, China
3 Aerogel Functional Nanomaterials Laboratory, Inner Mongolia University of Science & Technology, Baotou 014010, China
* Correspondence: furui14@mails.ucas.edu.cn (R.F.); shz15@tsinghua.org.cn (H.S.)
† These two authors contributed equally to this work.

1. Characterization

1.1. XRF and XRD

The major elements of the coal gangue were analyzed using an X-ray fluorescence (XRF) spectrometer (Zetium, PANalytical, Holland). XRD analysis was performed using an X-ray diffractometer (Empyrean, PANalytical, Holland) at 40 kV and 40 mA using Cu K_α radiation; at a scan rate of 10°/min.

1.2. Morphology and nanostructure

The microstructure of the SACABs was determined using scanning electron microscopy (SEM, Apreo 2, Thermo Scientific, America) aided by energy dispersive spectrum (EDS) analysis at an accelerating voltage of 10 kV and a working distance of 8.1 mm. The inner and outer surfaces of the samples were prepared for analysis by simply sticking sample slices on the sample holder using a carbon pad, followed by coating with platinum.

1.3. Surface wettability

The surface wettability of the SiO_2-Al_2O_3/agarose composite aerogel beads (SCABs) were measured using a contact angle measuring system (SZ-CAMC31, Xuanzhun, China). First, the SCABs were affixed to the sample holder using double-sided tape to ensure stability when the contact angles of the surfaces (internal and external) were measured. A droplet (2.5 μL) of deionized water was deposited on the surface of the SCAB hemisphere. At least four measurements were obtained for each sample.

1.4. Nitrogen physisorption measurements

Nitrogen physisorption measurements were performed using a surface analyzer (Quantachrome Ins, USA) at 77 K. Brunauer-Emmett-Teller (BET) analysis based on the amounts of N_2 absorbed at various relative vapor pressures (four points, $0.05 < p/p_0 < 0.3$, nitrogen molecular cross-sectional area=0.162 nm^2) was used to determine the specific surface areas [1]. The Barrett-Joyner-Halenda model was used to calculate the pore size distribution of the samples.

1.5. Thermal stability

The thermal degradation curves were obtained using thermogravimetric analysis (TGA, STA449F3, NETZSCH, Germany). A sample weight of approximately 2 mg was obtained. The sample was placed in a ceramic pan and heated in the temperature range of 30-700°C at a rate of 10 °C/min under atmospheric pressure.

1.6. Mechanical properties

The mechanical properties were analyzed using an HD-B609B-S mechanical property tester (China). All the SCABs were selected with an approximate size (diameter of approximately 3.0 mm). The compression stress–strain curves of the SCABs were determined at a compression speed of 2 mm/min. The SCAB testing ended when the deformation of the samples reached 70% (deformation value of SCABs = height variation / diameter × 100%).

1.7. Thermal transport properties

The temperature of the sample surface was evaluated using a FLIR-T620 infrared thermal imager (America) in red/blue color display mode. The samples were tested after 10 min of remaining at a constant surface temperature.

2. The major elements and phase of the raw coal gangue

Table S1. Elemental analysis of raw coal gangue by XRF.

Composition	SiO_2	Al_2O_3	MgO	CaO	Fe_2O_3	LOI[a]
(wt. %)	43.77	26.50	0.1	0.32	0.23	15.43

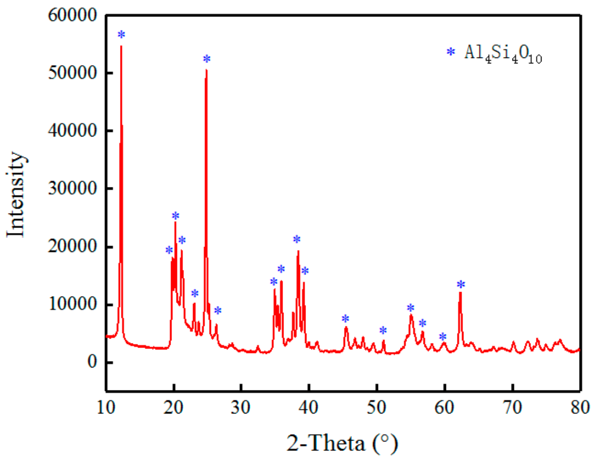

Figure S1. XRD patterns of the raw coal gangue.

3. Activation procedure of the coal gangue

The reaction rate of coal gangue was calculated by the following equations:

$$Reaction\ Rate = \frac{m'}{\mathrm{m} \times (1 - \mathrm{p})} \times 100\%$$

m is the original weight of coal gangue and m' is the weight of the insoluble solid from the reaction production of the sinter (coal gangue and Na_2CO_3) after mixing with 6 mol/L H_2SO_4 solution after adjusting pH=2 on a magnetic stirrer for 1 hour and p is the ignition loss of coal gangue.

To obtain the high reaction rate of coal gangue, three experimental parameters are studied, including weight ratio of coal gangue to Na_2CO_3 (m (Coal gangue)/m (Na_2CO_3)),

holding time, and calcination temperature. According to the literature, the gangue samples and Na_2CO_3 were prepared by screening the ground raw materials to a mesh of 100 respectively. The weight ratio of coal gangue to Na_2CO_3 was set as 10:4, 10:5, 10:6, 10:7, and 10:8 and then mixed evenly. Calcination times were set from 1.0 h to 3.5 h (each 30 min is a variable). The temperature was set to 650-950 °C (each 25 °C is a variable). The values of three experimental parameters are determined by single variable method [2].

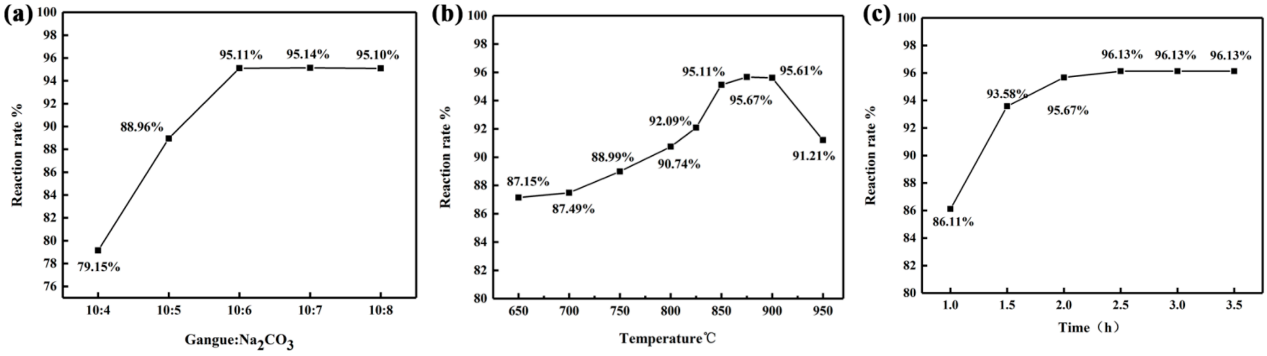

Figure S2. The reaction rate of coal gangue under different parameters.

The 5g of raw coal gangue was accurately weighed, and then mixed it with different masses of anhydrous Na_2CO_3 according to 10:4~10:8, subsequently calcined the mixture at 850°C for 2h. After the calcination reaction was completed, the activated coal gangue (ACG) was cooled at room temperature, and then excess dilute sulfuric acid was added. The residue were washed with deionized water after vacuum filtration, and then dried. The calculated reaction rates were shown in Figure S2 (a). By analyzing the data of ACG reaction rate, it could be concluded that when the mass ratio of coal gangue and Na_2CO_3 was 10:6, ACG was adequately decomposed.

The optimum ratio of coal gangue and Na_2CO_3 was 10:6 for mixing, the calcination time was 2h, and the calcination temperatures are 650°C, 700°C, 800°C, 825°C, 850°C, 875°C, 900°C, 950°C (The melting point of sodium carbonate is 851°C). As shown in Figure S2 (b), it could be seen that the reaction rate was lower at 650℃-825℃. At this time, the Na_2CO_3 did not reach the melting point. Hence, the coal gangue and Na_2CO_3 were solid-solid reaction. When the temperature reached 850 °C, the reaction rate was importantly increased to 94.72%. At this time, the Na_2CO_3 was mostly molten state leading to a solid-liquid reaction, so the reaction rate was improved. At 875℃-900℃, Na_2CO_3 was completely melted, and the reaction rate reached a maximum of 95.67% and remained basically stable. The optimal reaction temperature was 875℃.

When the reaction temperature was set to 875 °C and the ratio of coal gangue to Na_2CO_3 was 10:6, the reaction rate of coal gangue at different times was explored. The reaction rate remained basically stable after 2.5 h (Figure S2 (c)), indicating that the reaction was nearly complete and reached the maximum extent. The optimum calcination time was 2.5 h according to the analysis of the reaction rate.

In conclusion, the coal gangue was with Na_2CO_3 at mass ratio of 10:6 and the activated coal gangue was obtained calcined in 875 °C for 2.5 h. The main component of ACG is $NaAlSiO_4$ (Figure S2) that can be decomposed in acid solution to obtain the SiO_2 sol containing Al^{3+} (SSCA).

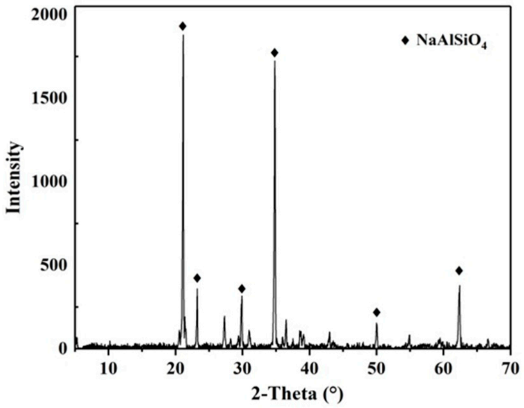

Figure S3. XRD patterns of the ACG.

4. The preparation of pure putty blocks (PPBs)

Putty powder (10.0 g) and water (4 mL) were mixed evenly to obtain putty paste. Next, the putty paste was shaped into a blocks of 7 mm thickness (the length and width were 30 mm and 25 mm respectively). Finally, the wet putty blocks were dried in oven at 50°C for 2 h to obtain PPBs.

5. The effect of adding water volume on gel

When 20 mL deionized water was added to the ACG, the wet gel formed rapidly about 0.6 h at room temperature, which resulted in the abundant residual substance of ACG that was not decomposed embed in the translucent gel as shown in Figure S4 (a). When the amount of water added exceeds 20mL (25-45mL), the gel time exceeded 1h. The solid particles in the suspension were obviously reduced (Figure S4 b). After filtration process, a little residue was remained on filter paper, and the obtained SSCA could be transformed into a transparent gel with the robust gel skeleton (Figure S4 c-e).

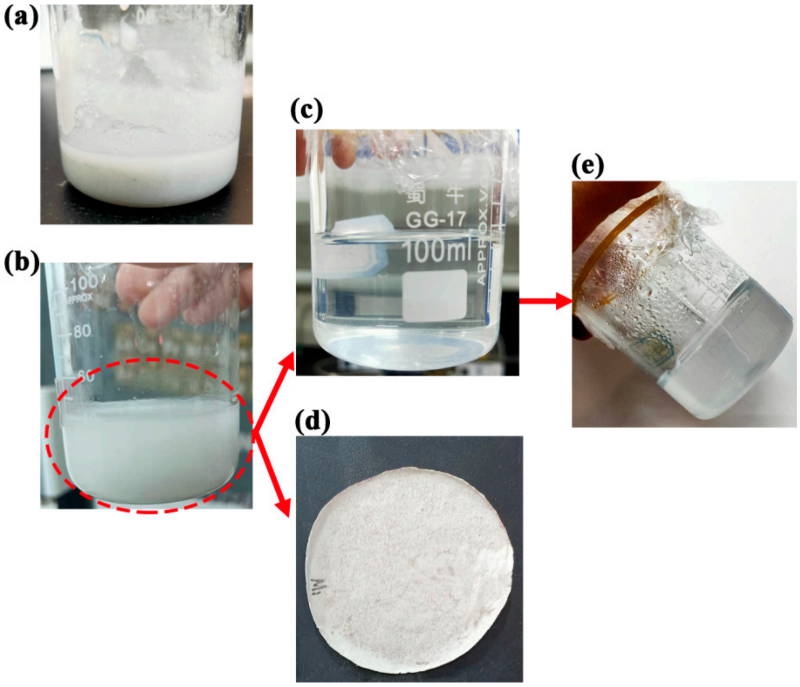

Figure S4. The effect of adding water volume on gel. (**a**) Wet gel formed after adding 20 mL water into ACG. (**b**) The suspension was formed after adding 40 mL water into ACG. (**c**) SSCA was obtained by filtering the suspension. (**d**) A little residue on filter paper. (**e**) The gel formed from SSCA.

6. Morphology photos of SCABs

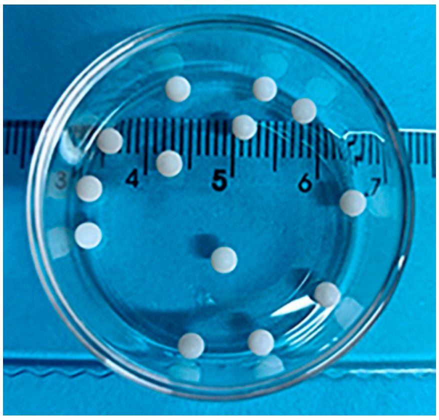

Figure S5. The photo of SCABs.

7. Infrared Spectroscopy

The FTIR spectrums of the AABs (native agarose aerogel beads) and unmodified and modified SCAB-4 are shown in Figure S6. The peak of samples at around 3392.35 cm^{-1} was ascribed to the antisymmetric stretching vibration of -OH bond. Peak of SCAB-4 at around 1050 cm^{-1} obviously wide than AABs, which is caused by existing Si-O-Si bonds of SCAB-4 [3]. In addition, the peaks at round 800 and 580cm^{-1} contributed to the Al–O and Si-O-Al bonds respectively in the SABs [2,3]. Therefore, these all mani-fested the successful preparation of SiO_2-Al_2O_3/ agarose composite aerogel using coal gangue. If the aerogels are modified with TMCS, bands at around 846 cm^{-1} corresponding to the deformation modes of Si-C bonds and the absorption peaks at around 2964 cm^{-1} corresponding to C-H bonds can be clearly observed from Figure S6 (a) [4]. The quite visible absorption bands of Si-C bond and C-H bonds show a Si-CH_3 for-mation, which is the main source of hy-drophobicity. Therefore, the SCAB-4 samples were successfully modified to introduce hy-drophobic groups.

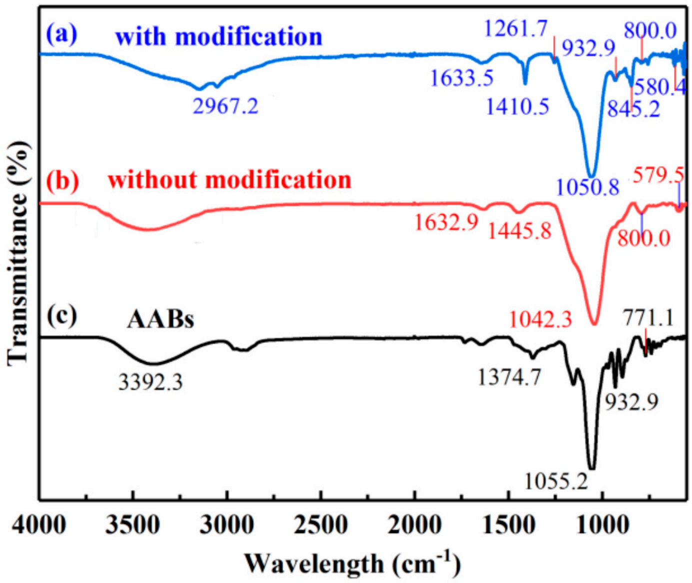

Figure S6. FTIR spectra of samples. (a) SCAB-4 with modification, (b) SCAB-4 without modification, (c) AABs.

8. XRD analysis of the SCAB

The XRD test was performed on the SCAB-4 synthesized and the result was illustrated in Figure S7. The signal of SCAB-4 was characterized by a wide shape between 20° and 27.5°. The X-ray diffraction spectra indicated that the obtained SCABs were amorphous, which was related to the destruction of the crystalline form in coal gangue during the preparation of the aerogel.

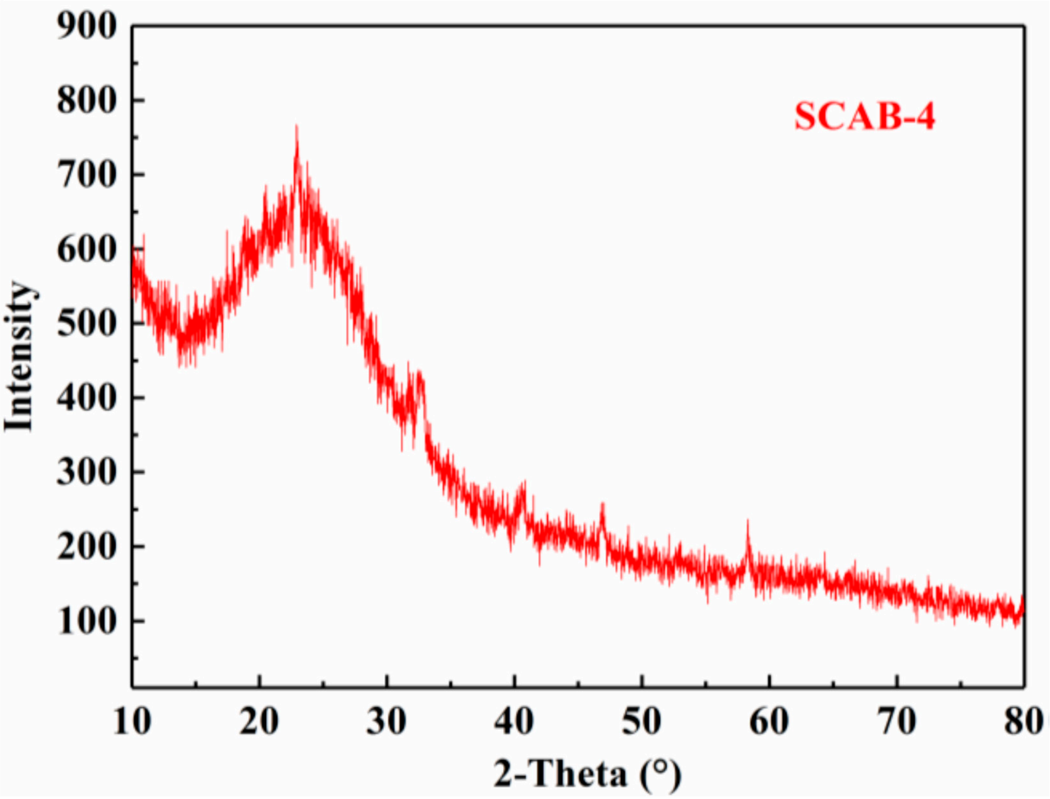

Figure S7. XRD patterns of the SCAB-4.

References

1. Sai, H.; Fu, R.; Xing, L.; Xiang, J.; Li, Z.; Li, F.; Zhang, T. Surface modification of bacterial cellulose aerogels' web-like skeleton for oil/water separation. *ACS Appl. Mater. Interfaces* **2015**, *7*, 7373-7381, doi:10.1021/acsami.5b00846.
2. Zhu, J.; Guo, S.; Li, X. Facile preparation of a SiO_2–Al_2O_3 aerogel using coal gangue as a raw material via an ambient pressure drying method and its application in organic solvent adsorption. RSC Adv. **2015**, 5, 103656-103661, doi:10.1039/c5ra20392a.
3. Shen, M.; Jiang, X.; Zhang, M.; Guo, M. Synthesis of SiO2-Al2O3 composite aerogel from fly ash: a low-cost and facile approach. J. Sol-Gel Sci. Technol. 2019, 93, 281-290, doi:10.1007/s10971-019-05204-y.
4. Hu, W.; Li, M.; Chen, W.; Zhang, N.; Li, B.; Wang, M.; Zhao, Z. Preparation of hydrophobic silica aerogel with kaolin dried at ambient pressure. Colloids Surf. A 2016, 501, 83-91, doi:10.1016/j.colsurfa.2016.04.059.
